# Filling Teeth

**Published:** 1872-03

**Authors:** C. R. Taft


### FILLING TEETH.

Read Before the Ohio State Dental Society, at the Sixth Annual Meeting, December, 5, 1871.

BY C. R. TAFT, D. D. S.

So much has been written and so much of the time of every Dental Society and Association consumed in discussions on the subject of Filling Teeth, that it would seem as if some apology were necessary for further inviting your attention to the subject, but since the Chairman of your Executive Board has given this as one of the topics for our consideration at this time, I have ventured to throw together a few thoughts under this head.

Conservative Dentistry stands to day in the front rank of the specialties of our profession. It is this which is claiming and receiving the attention of the best dental practitioners in our country. It is the practice of this more than any other one thing that raised our profession to its present proud position when compared with the place it occupied twenty-five or thirty years ago, and it is in the preservation of the natural teeth that we are benefiting more of our fellow men than in any other branch of our profession.

If this is so, why should we excuse ourselves from writing upon and discussing this all important subject in all its bearings, until we arrive at something like uniformity in our practice, and until all Dentists shall become good operators upon the natural teeth.

So long as we find so few good fillers, (and they are truly very few among the great number who have received or taken upon themselves the title of Dentist), so long as we have presented to us almost every day imperfect, not to say very bungling work upon the natural teeth. So long as we hear the cry of fillings coming out in one or two years, or perhaps as many months; we have abundant cause to discuss this subject, and see where the failure of so many lies.

I am aware of the difficulty of describing the manner of filling any given cavity in a tooth. To attempt a description of the filling of all the variously formed cavities found upon the different teeth, would be a task much beyond anything contemplated in this paper. But there are certain rules and principles in the filling of all the teeth, which should be observed, and which if carefully followed, good results may be obtained.

The cavity of the tooth to be filled should be so opened that every portion of the border can be distinctly seen and easily reached with the instruments used in excavating it, as well as those afterward employed in packing the gold. Where wedging will not accomplish this, a sufficient amount of the enamel and tooth bone must be sacrified to attain this end, the lost portions being of course restored in the operation of filling. The edges of the cavity after the removal of the decay, should be trimmed down until strong, regular and smooth borders are secured, against which the gold is to be packed. If the cavity is properly shaped it is the nice joining of the gold to the margins of the cavity that makes the perfect filling. The cavity should be so shaped as to have some under cutting or retaining points at its opposite sides, and these need not be either very large or deep, if the gold is securely packed into them. Adhesive gold should always be used where the best results are aimed at, and this thoroughly consolidated with a leaden mallet in the hands of an assistant when practicable. When contour fillings are made, and the gold built out beyond the walls of the cavity, every step of the operation of packing should be so performed that the gold would take as perfect a finish after consolidating each separate piece as it is capable of receiving, after the filling is completed.

The method some have of hastily cramming the cavity with the amount of gold they are able to get into it, leaving the ends protruding from the cavity, and afterwards consolidating these, forming a crust on the surface to receive a finish, while beneath the filling is yielding, and easily withdrawn from the walls of the cavity and loosened in the process of finishing, is a sure way of making an imperfect filling, and can not be too strongly condemned. The natural form of the tooth should always be restored in filling, and this, where there is any considerable loss of enamel and tooth bone, can only be done with adhesive gold. The rubber dam, if not used in excavating, which it is better to do, should as a rule always be applied before packing the gold, for only with absolute dryness can the best results be reached. When a cavity in a tooth is presented for filling, if on the approximal surface, the first thing to decide upon is the best mode of gaining free access to it, first to remove the decay and form the cavity, and afterwards introduce the filling. The wedge is here generally employed as a help, using two, one at the point of the tooth, where it exerts greatest power in separating the teeth, and afterwards introducing another next the gum. If sufficient space is not thus obtained, some portion of the enamel is cut away until the desired room is secured.

The next step is the removal of the decayed portion of the tooth, and the careful preparation of the cavity to receive the gold. It is in this latter operation that quite as many, or more, failures are made than in any other part of the work of filling a tooth. While the decayed and decaying portion of the tooth bone should as a rule be entirely removed, it is not bad practice many times to allow discolored and partially decayed bone to remain in the bottom of the cavity, where the decay approximates too closely to the nerve. If touched with creosote immediately before filling, it affords a much better covering for the nerve than any artificial capping. I have long since ceased to hunt for the nerve. Those who do can usually find it if they go deep enough. The bridging over of this softened bone in filling, is no very difficult matter, even when slight pressure in the direction of the nerve would give very perceptible pain. As the borders of the cavity are approached, the greatest care should be observed in removing all decay until firm and solid bone or enamel is reached. These should be trimmed down with the file or chisel until secure and regular margins are obtained. Many otherwise good fillings are lost, simply in saving thin and friable portions of enamel, against which it is impossible to pack the gold so as to make good joints, and in a little while we find these fillings have leaked; the enamel finally gives way and the filling drops out. The careful operator will always cut as little as is consistent with securing a good operation, and avoid as far as possible the exhibition of gold, but where the saving of the tooth and durability of the filling requires it, he should not hesitate to cut freely. In the case of the superior incisors, where the cavity is difficult of access, it is often, and I might say, generally best to cut away the enamel on the lingual surface, where it is usually more frail than on the labial surface, and excavate and fill the tooth through the opening thus formed. The decay being removed and the margins of the cavity thus carefully prepared, the next step is the formation of retaining points to secure the gold while being packed, and retain it when introduced. These may be formed with excavators, drills or very small chisels. It is best to have some undercutting at the base of the cavity, but this need not be extensive, as the excellent quality of the gold furnished now, and its cohesive properties, if perfectly packed, are such that very slight retaining points are sufficient to secure the filling in its place.

If the work thus far has been done without the use of the rubber dam, it should now be applied. It is the indispensable thing in securing the perfect packing of the gold into the cavity, and should never be dispensed with except perhaps in short operations for the superior teeth.

While nearly all of our Dentists assent to the use of the dam, and claim to use it sometimes themselves, it is probable that not one in twenty use it habitually. When the security from moisture, and the perfect freedom from anxiety on the part of the operator, is taken into account, and the excellent results attained are considered, it is a matter of astonishment and regret, that all pretending to make good gold fillings do not use it constantly. I am aware of the objections which many make to it. The difficulty of application; the mouth stretching, &c. The first comes, I apprehend, from those who have had very little experience in the use of it, and after one, two or three trials and as many failures, have gone back to stopping the mouth with napkins and bibulous paper, which is generally much more annoying to the patient than the application of the dam, if it is at all skillfully handled. The second objection amounts to nothing, as in the hands of one who has had any considerable amount of experience, it may be applied in from one to three minutes almost anywhere upon the teeth, and with much less discomfort to the patient than some other portions of the operation. We do not hesitate to cut the tooth though the bone should be excessively sensitive, nor should we discard the use of rubber, though there may be a little stretching and a little pain in applying it to the teeth of persons with small mouths. To those who aspire to the best results in filling, I say take up the dam and use it constantly, and the comfort with which you operate, and the results you accomplish, will abundantly repay for the little trouble and annoyance you may experience in first learning to use it. With the dam on and the edges nicely turned under, and the rubber firmly secured in place with ligatures or wedges between the teeth, where there may be a tendency for it to slip from some one or more of the teeth, and with the cavity prepared as before indicated, we are ready for the introduction of the gold, having wiped it thoroughly, dryed with cotton spunk or bibulous paper. The gold is started by driving a small pellet or narrow strip (if heavy gold is used) into a small pit at the base of the cavity, previously formed in the most accessible locality. If the cavity is broad and shallow, two or more of these pits may be formed, and when all are filled, spanning over from one to the other, covering the base of the cavity, and piece by piece adding on until the cavity is full, and a little more than the full form of the tooth restored, to allow for finishing, care being taken at every step to have the gold perfectly packed at the margins of the cavity, and to insure this there should be no portion of the borders that can not be plainly seen as the work progresses. That heavy numbers of gold now being extensively used by most of our best operators, makes the finest filling, I think there is little doubt, even very small cavities and fine fissures are most perfectly filled with it. It seems to adapt itself to the walls of a cavity more perfectly than the lower numbers of foil, and the integrity of the plug when completed, is undoubtedly better. Perhaps number 60 and 120 are most used, and with these as good or better fillings can be made than with any other form of gold at present furnished us. Soft, or non-adhesive foil, should seldom or never be used for filling teeth. There are few cases where a really good filling can be made with it, and the contour of the tooth restored at the same time if there is any loss of the enamel, and I may say none, where a better filling can not be made with adhesive gold. I will not deny that many of our best operators of the old school, made fillings twenty or thirty years ago with non-adhesive foil, that saved the teeth, but were their operations on the back teeth not made at a terrible sacrifice of the tooth substance, causing the teeth to appear like so many inverted wedges? And may it not be that the few of these very old and successful fillings that we see to-day, are found in teeth, that were at the time they were filled, in the very best possible condition to resist decay, for we all know how poor a filling will sometimes seem to save a tooth. If these old and careful soft foil fillers did succeed in saving very many teeth, who will say that they would not have succeeded infinitely better had they used the adhesive foil we are using to-day, for certainly they secured no such joining of the gold to the tooth and finish of the filling, as our operators are accomplishing at the present time with adhesive gold. To say nothing of their entire failure to restore any portion of the lost tooth beyond the margins of the cavity filled. No, the day of non-adhesive fillings is past. Its advocates will usually be found among those who aspire to making twenty fillings in a day, and who have the satisfaction of seeing three-fourths of them leaky and imperfect, out, or ready to drop out, at the end of the first year. For one I am not anxious to be counted among those extraordinarily rapid operators, and am quite content if I make from two to six or eight fillings in a day, taking the cavities as I find them in the mouths of my patients. I do not say that a greater number of fillings might not be made in a day, if only the small pits and fissures on the grinding surface of the molars and bicuspids are selected, but the man who claims to fill habitually twenty cavities in a day, is talking nonsense, and knows if he is honest, that his work when done is worthless.

But to drop this, let us take up the filling of a cavity on the posterior approximal surface of a molar or bicuspid, which is of every day occurrence, and usually considered among the more difficult kind of cavities to fill. After driving the wedge to secure the desired space, the enamel on the grinding surface should be cut through into the cavity beneath, (if it is not already broken through), and this cutting should be so extensively done as to admit of a perfect view of the bottom and outer and inner walls of the cavity. The decayed portions of the tooth removed and the margins trimmed down as heretofore indicated. We will allow that we find the walls of the cavity flaring outward, forming what is frequently called a spoon shaped cavity, a retaining groove should now be cut at the base of the cavity, and in it the pit or pits from which the gold is to be started. We next come to the opening of our cavity at the grinding surface where, if the walls are thick and strong, grooves may be cut to secure the filling at the top, but in most cases where the fissures give indication of moisture having penetrated them, and early decay is probable, it is better to cut them out, connecting them with the cavity proper, and form a pit at the end of the cutting. This being done, and the rubber dam in position, the cavity perfectly dry, first having been wiped out with creosote, we are ready for the filling. I have been in the habit of using for cases of this kind steel keys, which are readily formed for any particular case from the smooth portions of separating files, and cut to the desired width and length, and with the pliars given the exact curve of the tooth, or the form desired when the filling is completed. I now slip the key down between the teeth, and with a wedge behind it secure it firmly in place. When the cavity is very deep, a key sufficiently broad to come to the top of the tooth, might obstruct the view. I use a narrow key at the bottom, and after the gold is carried to the top of this slip in another in like manner, and proceed as before until the gold is built up as far as the grooves formed by cutting out the fissures. The latter are now filled with strips of heavy gold, (if heavy gold has not been used throughout), one end united to the filling proper, and the other carried over and driven into the pit formed at the end of the groove, after this is done the gold is built on until the contour of the tooth is restored. In this way the filling is securely anchored at the top, and if the borders have been carefully watched, we consider we have a good filling. The key I consider invaluable in filling this class of cavities, especially in small mouths, when it is difficult and many times impossible to bring the instrument used in packing on a line with the axis of the tooth, and where from the inclination of the instrument there is a tendency to drive the gold out of the cavity rather than against its walls. But with the key you are really filling a crown cavity, and have every opportunity of wedging the gold securely against the walls of the cavity, as if filling on the anterior surface of the tooth. With the use of the key the labor of finishing is materially lessened as the approximal surface seldom needs more than a little trimming at the borders and a few minutes use of the finishing tape.

Of the materials in use for filling teeth it will scarcely be questioned by any here present, that gold stands pre-eminently at the head, and save for temporary or test fillings, little else should be used. Oxychloride of zinc as a capping for exposed nerves and for filling the inner portion of very large cavities, is excellent, but as a filling for masticating purposes I believe it is considered a failure. The secretions found in most mouths acting upon it, and little by little it is washed away. Hill's stopping or rubber, has its place, and when put into a tooth where it is required to play no part in masticating, it makes a filling that will preserve a tooth for a long time. Of that dark visaged imp Amalgam, which finds its way into so many mouths when so few use it, what shall I say, should it ever be used or not? That it has and is, saving some teeth, we must all admit, but in the manner in which it is generally used, I doubt not it has caused the loss of thousands more than it ever saved. That in the hands of a good and careful operator who will give the requisite time to the preparation of the cavity, and the finishing and burnishing down the edges of the filling, which is an after operation, and which as a rule is never done at all. I doubt not fillings can be made which will save the teeth for a time and prove of service; but the blackened appearance which it almost always assumes and imparts to the tooth renders it wholly unfit as a filling in the front portion of the mouth, and exceedingly objectionable anywhere. One great objection to amalgam is that the fillings made with it are usually made at a price that would not one-half pay a good operator for properly preparing the cavity for any kind of a filling. If the proper fee was charged for making as good amalgam fillings as can be made, we would not often be asked to use it, for the patient would almost invariably be willing to pay the additional price and have the tooth filled with gold. I have never seen very many good and well finished amalgam fillings. The best, or among the best I ever saw, were put in under protest by that excellent operator, our present worthy president. They were large contour fillings on the molars, beautifully formed and nicely finished, look well, and are doing good service to-day. But for these fillings he received a price for which many of us put in quite large gold fillings.

